# A Feature Extraction Algorithm of Brain Network of Motor Imagination Based on a Directed Transfer Function

**DOI:** 10.1155/2022/4496992

**Published:** 2022-02-28

**Authors:** Shuang Ma, Chaoyi Dong, Tingting Jia, Pengfei Ma, Zhiyun Xiao, Xiaoyan Chen, Lijie Zhang

**Affiliations:** ^1^College of Electric Power, Inner Mongolia University of Technology, Hohhot 010080, China; ^2^Intelligent Energy Technology and Equipment Engineering Research Center of Colleges and Universities in Inner Mongolia Autonomous Region, Inner Mongolia, Hohhot 010051, China

## Abstract

Aiming at the feature extraction of left- and right-hand movement imagination EEG signals, this paper proposes a multichannel correlation analysis method and employs the Directed Transfer Function (DTF) to identify the connectivity between different channels of EEG signals, construct a brain network, and extract the characteristics of the network information flow. Since the network information flow identified by DTF can also reflect indirect connectivity of the EEG signal networks, the newly extracted DTF features are incorporated into the traditional AR model parameter features and extend the scope of feature sets. Classifications are carried out through the Support Vector Machine (SVM). The classification results show the enlarged feature set can significantly improve the classification accuracy of the left- and right-hand motor imagery EEG signals compared to the traditional AR feature set. Finally, the EEG signals of 2 channels, 10 channels, and 32 channels were selected for comparing their different effects of classifications. The classification results showed that the multichannel analysis method was more effective. Compared with the parameter features of the traditional AR model, the network information flow features extracted by the DTF method also achieve a higher classification effect, which verifies the effectiveness of the multichannel correlation analysis method.

## 1. Introduction

Brain-computer interface (BCI) technology can realize direct information interactions between brains and the outside world. Among the input signals of BCI systems, the motor-imaging EEG signals have been widely used in the BCIs as spontaneous EEG signals. As well known, the motor imaging BCI has an important application value in the medical field. For example, it can be used for rehabilitation training of patients with motor dysfunction [1] or for controlling wheelchairs and robotic arms through motor imaging [2, 3]. The function can bring a great convenience to the patients' lives. In addition, BCI technology also has a wide range of applications in human's daily lives: robot control, smart home, computer games, and so on [4–6].

When performing motor imagination, it usually causes event-related desynchronization (ERD) and event-related synchronization (ERS) phenomena in the brain [7]. Thus, different features can be extracted according to the different EEG signals generated by different motor imaginations so that the features can be further used to identify the types of EEG signals of different motor imaginations. Commonly used feature extraction algorithms include co-space mode, wavelet transform, autoregressive model, power spectrum estimation, and other algorithms [8]. These algorithms generally extract features from a single channel of EEG signal and less consider the correlation between different channels. At present, more and more researches use the method of correlation analysis to identify the information flow between different channel signals in the motor imagery EEG signal to construct a brain network and extract network features.

The neural connectivity of the brain networks is usually investigated in three aspects: structural brain network, functional brain network, and causal brain network [9]. Structural brain networks are the physiological connections between different neurons, which represent the physiological synaptic structures of the brain. Functional brain networks represent the statistical-based associations of biological neural networks and do not consider the flow of information and causal connections between nodes. Causal brain networks reflect the information transmission and interaction between different areas of the brain and is a directed network. In an effective brain network, effective connectivity shows the direction and intensity of information flow between different brain regions [10].

Some studies have used the connectivity between different areas of the brain to analyse motor imagery EEG signal. Daly et al. used modal decomposition to analyse the phase synchronization between the Intrinsic Mode Functions (IMF) of all channels of EEG data and built the brain's information connection networks [11]. The clustering coefficient is used as the feature of the connectivity between different channels to classify the motor imagery EEG signals, and good experimental results are obtained. Billinger et al. calculated the connectivity between various brain regions through the direction transfer function and used this as a feature vector to determine the category a motor imaging EEG signal belongs to. It is proved that the motor imagery EEG signal can be classified by the direction transfer function [12]. Li et al. proposed to use the partial directional coherence (PDC) algorithm to model the motor imagery causal effect networks and used the network parameters as features to realize the classification of the left- and right-hand motor imagery EEG signals [13]. Luo et al. proposed a feature extraction method combining brain function network and sample entropy [14]. In the method, the brain function network is constructed separately for the left and right hemispheres of the brain. The node degree and average clustering coefficient of the network are used as the characteristics of the brain function network. The new features are combined with the characteristics of sample entropy; an improved classification effect was achieved.

The aforementioned methods commonly focused on one aspect of the networks' properties and tried to find the unique informative feature for motor imagery EEG signal classifications. However, to the knowledge of updated neurophysiology, the information transmission often involves the various emerging properties coming from the complex brains' networks. The combinations of different network features were seldomly performed and investigated. This paper studies the use of the combination of AR parameters and Directed Transfer Function (DTF) algorithm to identify the connectivity and information flow between different channels and different brain regions in motor imagination. As known, the network information flow identified by the traditional method AR model can only represent the direct connectivity of the EEG signal network, which shows a lack of network structure features. Considering that the network information flow identified by DTF can also reflect indirect connectivity of the EEG signal networks, the newly extracted DTF features are incorporated into the traditional AR model parameter features and the scope of feature sets is extended. At the end, classifications are carried out through the Support Vector Machine (SVM). The classification results show the enlarged feature set can significantly improve the classification accuracy of the left- and right-hand motor imagery EEG signals. The DTF does not require a priori knowledge about network connectivity and is highly resistant to noise, which makes the method highly appropriate to use even in an environment with common noise. Additionally, for frequency analysis, the DTF is also resistible to constant phase disturbances and can be used even with an environment of attenuation of electrode signals and volume conduction effect, as known to be common in EEG signals.

## 2. Materials and Methods

### 2.1. AR Model

The autoregressive (AR) model belongs to a stationary time series model. The basic idea is to use an autoregressive process to fit EEG signals so that fewer parameters can be used to reflect more direct connective information [15]. The AR model is an all-pole model. In the time series, the information at the next moment can be weighted by the information at the previous moment and the current information. The time series of EEG data can be best fitted by selecting the appropriate order, which can be expressed by the following difference equation [16]:

(1) $$\begin{array}{c} x\left(n\right)=-\sum_{k=1}^{p}a_{k}x\left(n-k\right)+\varepsilon \left(n\right). \end{array}$$

In the formula, *ε*(*n*) represents white noise with a mean value of zero and a variance of *σ*^2^, *p* is the order, and *a*_*k*_ is the coefficient of the model.

When calculating AR model parameters, Burg algorithm is a commonly used algorithm. The algorithm is an autoregressive power spectrum estimation method, which can be estimated and calculated with less data, and the estimation result is very close to the true value. However, when this algorithm is used to process high-order models and the data is long, the estimation precision becomes lower, and the phenomenon of spectral line shift and spectral split may occur [17].

### 2.2. Directed Transfer Function

The DTF is used to determine the direction of information transfer between different variables in multivariable signals and show a good robustness. It can especially correctly identify the direction of information transfer in multivariable signals with a noisy. Therefore, the method is often used to determine the direction of information flow between time series in order to identify the direction of information flow of EEG signals in different channels and different brain sections.

Let vector *S*(*t*)=[*s*_1_(*t*), *s*_2_(*t*),…,*s*_*N*_(*t*)]^*T*^ denote *N*-channel EEG signals at time *t*. Fit the data set *S* through a multivariate autoregressive model [18]:

(2) $$\begin{array}{c} S\left(t\right)=\sum_{k=1}^{p}\Lambda_{k}S\left(t-k\right)+E\left(t\right). \end{array}$$

In (1), *E*(*t*)=[*e*_1_(*t*), *e*_2_(*t*),…,*e*_*N*_(*t*)]^*T*^ represents a white noise vector with a zero-mean white noise, and Λ_*k*_ is the *N* × *N* matrix of model coefficients. The notation *p* is the order of the model, which can be calculated by the AIC criterion. In order to study the nature of its frequency domain, the above formula can be transformed to the frequency domain [19]:

(3) $$\begin{array}{c} S\left(f\right)=\Lambda^{-1}\left(f\right)E\left(f\right)=H\left(f\right)E\left(f\right). \end{array}$$

Among them, *f* denotes a specific frequency and *H* (*f*) is the transfer matrix of the system from a white noise to the network response. Suppose all the outputs of the networks come from the white noise, which are independent from each channel. Thus, one element of *H*(*f*) represents the connections between the *j*th input and the *i*th output of the system. The transfer matrix is defined as

(4) $$\begin{array}{c} H\left(f\right)=\Lambda^{-1}\left(f\right)={\left(\sum_{k=0}^{p}\Lambda_{k}e^{-j2\pi f\Delta tk}\right)}^{-1},\Lambda_{0}=-I, \end{array}$$

where *I* is an identity matrix and Δ*t* is the time interval between two samples. A normalized form of *H*(*f*), named as DTF, is often used to determine the relationship between two signals in all signals of a network as [20]

(5) $$\begin{array}{c} \gamma_{ij}^{2}\left(f\right)=\frac{{\left|H_{ij}\left(f\right)\right|}^{2}}{\sum_{m=1}^{N}{\left|H_{im}\left(f\right)\right|}^{2}}, \end{array}$$

where *γ*_*ij*_(*f*) describes the ratio between the inflow from signal *j* to signal *i* and all inflows to signal *i*. Naturally, the value of DTF elements is in the interval of [0,1]. If *γ*_*ij*_(*f*)  = 1, it means that all of the information in signal *i* is composed of information from signal *j*. If *γ*_*ij*_(*f*)  = 0, it means that there is no information flowing into signal *i* in signal *j*.

### 2.3. Support Vector Machine

Support vector machines (SVM) have a wide range of applications in pattern recognition. Therefore, SVM can be applied to the situation where the input feature vector is both linearly separable and nonlinearly separable. When the problem of pattern recognition is linearly separable, the main idea is to find a classification hyperplane to maximize the feature distribution spacing on both sides of the hyperplane. In the nonlinear separable case, the basic theory is to map the feature vector to a high-dimensional space through nonlinear transformation. Then, the classification hyperplane is obtained so that the input feature vector becomes linearly separable. Thus, this hyperplane maximizes the distance between the two types of feature distributions [21].

Set the sample set as (*x*_*i*_, *y*_*i*_), where *i*=1,2,…, *L*, *x* ∈ *R*^*N*^. The interval of *y* is between {−1, 1}. When the samples are linearly separable, the positive and negative sample sets will be separated. The constraint conditions for the classification of positive and negative sample sets are expressed by the following formula [22]:

(6) $$\begin{array}{c} y_{i}\left[\left(w\cdot x_{i}\right)+b\right]-1\geq 0,i=1,2,\cdots L, \end{array}$$

where *w* is the normal vector of the classification surface and *b* is the classification threshold.

For linearly separable training samples, the classification function is

(7) $$\begin{array}{c} f\left(x\right)=\mathrm{sgn}\left(w\cdot x+b\right). \end{array}$$

In (7), sgn denotes the symbolic function. For nonlinear problems, it is necessary to map it to a high-dimensional linearly separable space through an appropriate kernel function. Transform the data from the original space to a high-dimensional feature space. Then, the linear classification after the nonlinear transformation can be accomplished. At this time, the corresponding discriminant function becomes [21]

(8) $$\begin{array}{c} f\left(x\right)=\mathrm{sgn}\left(\sum_{i=1}^{l}a_{i}y_{i}K\left(x_{i}\cdot x\right)+b\right), \end{array}$$

where *l* is the number of support vectors, *a*_*i*_ is a Lagrangian multiplier, and *K*(*x*_*i*_ · *x*) is a kernel function.

Choosing different kernel functions has an important influence on the classification effect of SVMs. Through experimental tests, SVMs with linear kernel functions have achieved very good results in pattern classification of motor imagery EEG signals. In the following calculations, the average classification accuracy rate is obtained through the tenfold cross-validation and multiple calculations.

### 2.4. Data Collection

**Figure 1:**
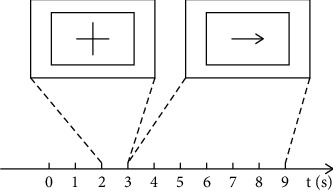
The experimental sequence diagram. The first two seconds are in an idle state. As a reminder for starting motor imagery of the left or right hand, a prompt symbol appears on the screen during the 2nd to the 3rd second. After that, an arrow to the left or right is displayed on the screen in the 3rd to the 9th second.

The acquisition of motor imaging EEG signals uses a 32-channel EEG equipment from BP Inc. in Germany. The 32 channels are all electrodes of the 32-channel EEG cap distributed according to the International 10–20 System Standards. Before the experiment, a conductive paste was injected into the electrodes of an EEG cap so that the electrode impedance was below 5 kilohms. Then, the sampling frequency was adjusted to 500 Hz. The experiment process is carried out in a quiet environment. The signal acquisition timing diagram is shown in Figure 1. The display interface is blank within 0 to 2 seconds. During that period, a subject relaxes and does not engage in thinking activities. Later within 2–3 seconds, a prompt symbol appears on the display interface to remind the subject to start imagining the movement of the left or right hand. In the future 3 to 9 seconds, an arrow appears on the display interface with a left or right direction. At this time, the participant imagines the movement of the left or right hand from the difference in the direction of the arrow. The experiment was divided into 4 groups, and each group performed 20 times of motor imagery. There are 7 subjects participating in the experiment, all around 25 years old and in good health. Eighty sets of data were collected for each person.

## 3. Results and Discussion

In the collected 32-channel EEG signals, 10 channels of EEG data of F3, F4, C3, C4, Fz, Cz, FC1, FC2, FC5, and FC6, which are in or near to the motor brain area, are selected for processing. The DTF is used to identify the connectivity between different channels. The frequency components of 10 Hz, 15 Hz, 20 Hz, 25 Hz, and 30 Hz are calculated for the motor imagery EEG signals. Initially, we have performed the Butterworth filtering of the primitive EEG data and then used the filtered data for the further feature extractions. However, for most data, the classification correct ratio went down 5% than that of unfiltered data does. Since the filtering effect of EEG data is not satisfying, the paper directly used the unfiltered data for the further feature extraction and task classifications. The DTF values between each electrode data is used as features, and then the aforementioned SVM is used for classifications. Then, the training sets (50% of the total data) and test sets (50% of the total data) were randomly selected and tenfold cross-validations were performed. The average classification accuracy rate is shown in Table 1.

As can be seen from the classification results, there are certain differences in the classification results of brain network characteristics of EEG signals with different frequencies in all subjects' motor imagination EEG signals. For the EEG signals of different subjects, the classification accuracies also show many individual differences. Subject 4 shows the highest classification accuracies, while subject 6 shows rather low classification accuracies for each frequency. Most subjects' motor imagination EEG signals reach the highest classification accuracy at 25 Hz and 30 Hz frequencies. Therefore, the DTF network features of the EEG signals calculated for 30 Hz were fused with the parameter features of AR model. The average classification accuracy both before and after feature fusion were shown in Table 2:

It can be seen from the classification results that for almost all subjects (except subject 1), the recognition results have been improved significantly after DTF feature fusion. For subject 1, the recognition accuracy of the fused features is lower than that of AR even if it is still higher than that of DTF.

Furtherly, all the 32-channel EEG signals were used to identify the connectivity between each channel. Then, the DTF values were also involved as features to classify the different tasks. The average classification accuracy rate is shown in Table 3.

It is easy to be found that the classification accuracies have been greatly improved with the involvement of more channel signals. It also shows that in the processes of motor imagination, there emerges more connectivity of information flow in a larger area of the brains. The longer distant connectivity may contribute significantly to classifying the different tasks.

For the 32-channel EEG signals, the DTF network features of the EEG signals calculated for 30 Hz were also fused with the parameter features of AR model. The average classification accuracies for the three methods were shown in Table 4.

Comparing Table 4with 2, we can see the classification accuracies have been significantly increased from 10-channel signals to 32-channel signals. It can also be found that the fused feature extraction method achieved higher accuracies than AR or DTF did in most cases, except for some few cases of subjects 2, 5, and 6. For example, the classification accuracies of AR + DTF are lower than those of AR for subjects 2 and 5, while the classification accuracies of AR + DTF are lower than those of DTF only for subjects 6. The exception cases can be explained by the individual performances of different subjects.

It can be seen from the experimental results that for the 10-channel EEG signal and the 32-channel EEG signal, the classification accuracy after the fusion of the single-channel feature and the network feature has been improved. The combination of direct network information flow and indirect network information flow is used when extracting network features. Consider not only the interaction information between neighbouring electrodes, but also the interaction information between electrodes that are farther apart. The single-channel features extracted by traditional feature extraction algorithms are combined with the network features constructed in this paper. The fused features improve the classification accuracy of motor imagery EEG signals, especially in the case of data analysis with few channels, the recognition accuracy of the fusion method is significantly improved.

**Figure 2:**
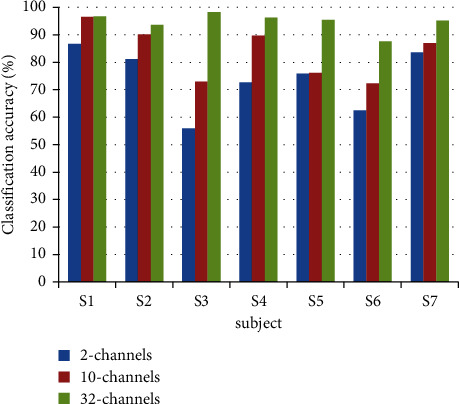
The average classification accuracies with the AR model features from the 7 subjects. The EEG signals of 2 channels, 10 channels, and 32 channels are, respectively, selected for processing; then, the AR model is used to extract the features. After that, the classifications are performed by the SVM, and the average classification accuracy rates of each group of data are calculated here.

**Figure 3:**
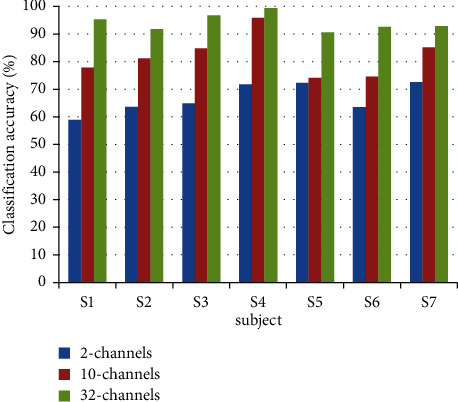
The average classification accuracies with the DTF features from the 7 subjects. The EEG signals of 2 channels, 10 channels, and 32 channels are selected for processing; then, DTF is used to extract network information flow characteristics. After that, the SVM is used for classification, and the average classification accuracies of each group of data are calculated here.

In order to verify the influence of the number of channels selected for EEG signals on the processing results, the EEG signals of 7 subjects were processed using the main C3 and C4 channels of the left and right hemispheres of the brain, as well as the abovementioned 10-channel and 32-channel EEG signals. The features are extracted through the AR model, and the average classification accuracy is shown in Figure 2. The characteristics of network information flow are extracted through DTF, and the average classification accuracy is shown in Figure 3.

As can be seen from Figures 2 and3, for the features extracted by the AR model and the features extracted by the DTF algorithm, as the number of selected channels increases, the classification accuracy rate gradually increases. It shows that when performing left- and right-hand movement imagination, the brain areas exhibiting significant EEG signal correlations outside of the areas of C3 and C4 electrodes, that is, the EEG signals of other channels, also have shown more wider information flows between traditionally observed brain areas and other neighbouring channels. As the number of selected channels increases, the classification accuracy of EEG signals can be improved. When the number of channels is large, the features extracted by analysing the brain network information flow method also achieve better results compared with the traditional methods, and the classification accuracy of the motor imagery EEG signal is improved after the feature fusion.

## 4. Conclusions

In the paper, the features of motor imagery EEG signals are extracted from the connective characteristics of brain networks. The network information flows between different channels and different brain regions are identified and the extracted brain network features are used for further task classification. The network features are then combined with the traditional AR model parameter features. The new feature combination improves the classification accuracies for the left- and right-hand motor imagery EEG signals. In addition, with more channel signals involved, the classification accuracies of motor imagery EEG signals have also been improved significantly. The result shows that there is a wide range of network information flows between different channels and different regions in the brains. These network information features can contribute to the classification accuracies of motor imagery EEG signals.

Accurate identification of network structure requires a large amount of multichannel neuronal response data with a high temporal and spatial resolution. As known, the data with electroencephalography (EEG) usually has a satisfied temporal resolution but has a low spatial resolution. Therefore, the number of the commands represented by motor imagery EEG signals is limited in view of the low spatial resolution of EEG signals. To overcome the drawback, some other existing measurement methods, such as functional Magnetic Resonance Imaging (fMRI) and Invasive Electrode Implantation (IEI), which have higher spatial resolutions may be the promising methods to merge the gap of limited command representation. Additionally, the other essential properties of the biological neurons are nonlinear dynamic and neuronal plasticity. Currently, there are few effective network structure reverse identification methods, which can accurately model and adapt this nonlinear and time-variant dynamic relationship. Further, a nonlinear time-variant method is required for extracting the precious network information flow features in neural networks. With the development of complex network researches, some topological characteristics of complex networks, for example, node degree and its distribution characteristics, degree correlation, agglomeration degree and its distribution characteristics, shortest distance and its distribution characteristics, node betweenness and its distribution characteristics, have already become the intriguing potential features for improving the classification accuracies for motor imagery EEG signals. In addition, the data collected in this manuscript may be subjected to some degree of noises and disturbance. A fuzzy preprocessing method [23] can be used to improve the robustness of the proposed fusion feature extraction method for analysing motor imagery EEG signals. The excellent performance of the fuzzy similarity approach proposed in [23] has been verified and confirmed for assessing the mechanical integrity of steel plates [24].

## Figures and Tables

**Table 1:** The average classification accuracy rate of the 10-channel brain network feature constructed by the DTF features. For the 10-channel EEG signals of 7 subjects, the DTF values were calculated using frequency bands of 10 Hz, 15 Hz, 20 Hz, 25 Hz, and 30 Hz, respectively. The DTF value between every two channels is used as a feature and classified by SVM.

Subject	10 Hz	15 Hz	20 Hz	25 Hz	30 Hz
1	73.7	72.2	75.5	76.4	**77.8**
2	75.5	79.1	80.6	81.2	**80.5**
3	80.5	83.5	84.2	84.8	**82.4**
4	83.1	89.3	92.6	93.8	**95.8**
5	73.9	74.6	73.5	74.1	**72.4**
6	65.7	67.4	70.6	72.8	**74.5**
7	80.8	83.4	82.8	83.7	**85.1**

The bold values indicate the best accuracies corresponding to the selected frequency of 30 Hz.

**Table 2:** The average classification accuracies after the fusion of AR model parameters and DTF brain network features. For 10-channel EEG data, the AR features, DTF features, and AR plus DTF fusion features are used to classify the different patterns by the SVM; then, the classification accuracies are calculated as follows.

Subject	AR	DTF	AR + DTF
1	96.5	77.8	**94.2**
2	90.1	81.2	**90.2**
3	72.9	84.8	**85.1**
4	89.7	95.8	**97.1**
5	76.1	74.1	**77.8**
6	72.3	74.5	**80.8**
7	86.9	85.1	**91.3**

The bold values indicate the best accuracies corresponding to the fused algorithm AR+DTF.

**Table 3:** The average classification correct rate of DTF feature extraction. For the 32-channel EEG signals of the 7 subjects, the DTF values were calculated using frequency bands of 10 Hz, 15 Hz, 20 Hz, 25 Hz, and 30 Hz, respectively. The DTF values between every two channels are used as features that are classified by the SVM.

Subject	10 Hz	15 Hz	20 Hz	25 Hz	30 Hz
1	89.7	91.2	92.1	93.6	**95.2**
2	83.8	88.8	90.8	90.8	**91.4**
3	93.7	95.3	96.1	96.5	**96.5**
4	95.6	97.2	98.7	99.1	**99.1**
5	90.8	91.3	91.1	90.8	**89.9**
6	90.5	93.1	93.3	91.6	**92.5**
7	83.8	88.8	89.7	90.8	**93.1**

The bold values indicate the best accuracies corresponding to the selected frequency of 30 Hz.

**Table 4:** The average classification accuracies after the fusion of AR model parameters and DTF brain network features. For 32-channel EEG data, the AR features, DTF features, and AR and DTF fusion features are used to classify through SVM, and the classification accuracies are calculated as follows.

Subject	AR	DTF	AR + DTF
1	96.7	95.2	**97.1**
2	93.7	91.8	**92.0**
3	98.2	96.8	**98.3**
4	96.3	99.3	**99.5**
5	95.5	90.6	**95.0**
6	87.6	92.5	**91.5**
7	95.2	92.8	**95.8**

The bold values indicate the best accuracies corresponding to the fused algorithm AR+DTF.

## Data Availability

The EEG data of the 7 subjects used to support the findings of this study are available from the corresponding author upon request. The contact information is dongchaoyi@hotmail.com.
